# Single-cell RNA-seq reveals distinct injury responses in different types of DRG sensory neurons

**DOI:** 10.1038/srep31851

**Published:** 2016-08-25

**Authors:** Ganlu Hu, Kevin Huang, Youjin Hu, Guizhen Du, Zhigang Xue, Xianmin Zhu, Guoping Fan

**Affiliations:** 1 School of Life Sciences and Technology, Tongji University, Shanghai 200092, China; 2 Department of Human Genetics, David Geffen School of Medicine, University of California Los Angeles, Los Angeles CA 90095, USA; 3 Translational Center for Stem Cell Research, Tongji Hospital, Department of Regenerative Medicine, Tongji University School of Medicine, Shanghai 20065, China; 4 Shanghai Pulmonary Hospital, Tongji University School of Medicine, Shanghai 200433, China; 5 Wuxi Medical School, Jiangnan University, Wuxi City, Jiangsu Province, China

## Abstract

Peripheral nerve injury leads to various injury-induced responses in sensory neurons including physiological pain, neuronal cell death, and nerve regeneration. In this study, we performed single-cell RNA-sequencing (scRNA-seq) analysis of mouse nonpeptidergic nociceptors (NP), peptidergic nociceptors (PEP), and large myelinated sensory neurons (LM) under both control and injury conditions at 3 days after sciatic nerve transection (SNT). After performing principle component and weighted gene co-expression network analysis, we categorized dorsal root ganglion (DRG) neurons into different subtypes and discovered co-regulated injury-response genes including novel regeneration associated genes (RAGs) in association with neuronal development, protein translation and cytoplasm transportation. In addition, we found significant up-regulation of the genes associated with cell death such as *Pdcd2* in a subset of NP neurons after axotomy, implicating their actions in neuronal cell death upon nerve injury. Our study revealed the distinctive and sustained heterogeneity of transcriptomic responses to injury at single neuron level, implicating the involvement of different gene regulatory networks in nerve regeneration, neuronal cell death and neuropathy in different population of DRG neurons.

In adult mammals, peripheral nerve injury triggers profound transcriptional changes in the soma of neurons that lead to maladaptive changes such as hyperexcitability and cell death or adaptive changes such as nerve regeneration and functional recovery^1^,^2^,^3^. Microarray and RNA-seq studies on bulk sensory neurons (quite often mixed with intact neurons and glial cells in the ganglion) have uncovered hundreds of axotomy-injury response genes in DRG^4^,^5^. However, analysis of bulk samples is not capable of distinguishing different cell types which undergo either cell death and neuropathic pain or alternatively nerve regeneration and functional recovery due to injury-evoked transcriptional changes.

DRG comprises several subtypes of sensory neurons with different functions^6^. These sensory neurons have been classified into distinct subtypes based on their cell-body diameters and gene expression patterns. For example, nonpeptidergic nociceptors (NP) are small unmyelinated neurons expressing purinergic receptor P2X ligand gated ion channel 3 (*P2rx3*); peptidergic nociceptors (PEP) are medium-sized lightly-myelinated neurons expressing calcitonin gene-related peptide (*Calca*); mechanoreceptors and proprioceptors are large diameter myelinated (LM) neurons with the expression of both neurotrophic receptor tyrosine kinase 2 (*Ntrk2*) and neurotrophic receptor tyrosine kinase 3 (*Ntrk3*)^7^. Under the physiological condition, DRG neurons were recently categorized into different subtypes by single-cell analysis of their transcriptomic patterns^8^,^9^. Furthermore, the injury responses vary among different subtypes of DRG sensory neurons after peripheral nerve transection^10^,^11^. For example, neuropathic pain is usually absent in the injured large myelinated neurons which can survive after axotomy^12^,^13^,^14^. In contrast, damage of cutaneous sensory neurons including NP and PEP typically results in small fiber neuropathy, a prevalent neurological disorder associated with neuropathic pain and neuronal cell death^15^,^16^. However, the transcriptomic perturbation in post-traumatic DRG neuron subtypes has not yet been characterized at single-cell level.

In the present work, we performed SNT in adult mice to generate an injury model of DRG neurons. After single-cell RNA-seq (scRNA-seq) analysis, we distinguished the heterogeneous injury-induced transcriptional alteration in subtypes of DRG neurons. We found that differentially regulated genes are functionally correlated with the fates of neuron subtypes after injury including shared up-regulation of regeneration associated genes (RAGs) in all subtypes of neurons (i.e., LM, PEP and NP) as well as preferential induction of cell death related genes in small NP neurons.

## Results

### Transcriptional profiles of peripherally axotomized DRG neurons via scRNA-seq

**Figure 1:**
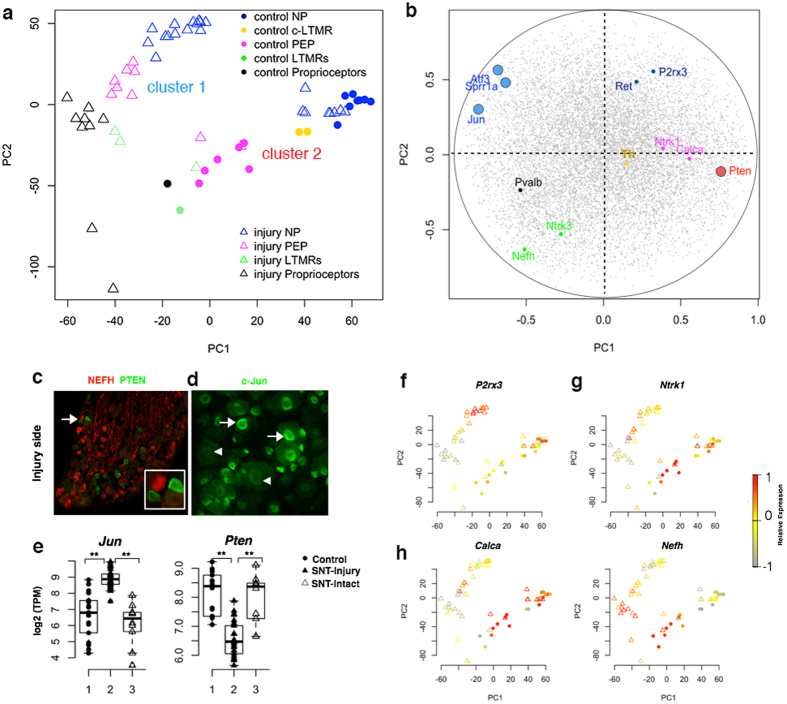
Single-cell RNA-seq analysis of sensory neurons with differential injury response after sciatic nerve transection. (**a**) Unbiased PCA analysis of mRNA transcriptome in individual sensory neurons via single-cell RNA-seq analysis. Individual DRG neurons were annotated according to their different expression patterns of marker genes in Fig. 1f,i and Supplementary Fig. 4b–e. NP, nonpeptidergic nociceptors; PEP, peptidergic nociceptors; and LM, large myelinated sensory neurons. (**b**) Genes characterized in each cluster and DRG subtype based on their correlation with PC1 and PC2 (Each gray point refers to a gene. Known regeneration/inhibitory associated genes in cluster 1 and 2 are highlighted in blue and red circle respectively. Known neuronal subtype markers are highlighted in color point.). (**c**) Immunofluorescence confirming intact cells of SNT-side L5 DRG with positive PTEN expression. (**d**) Immunofluorescence showed normal nuclei (arrow head) in intact c-Jun^−^ neurons compared to peripherally migrated nuclei (arrow) in injured c-Jun^+^ neurons in SNT-side L5 DRG. (**e**) Representative gene expression in control, SNT-injury and SNT-intact DRG neurons. **P < 0.01, one-way ANOVA. (**f–i**) Relative expression level of DRG neuron type-specific marker was mapped back to each sample. Color key represents normalized gene expression with the highest expression marked red and the lowest marked gray.

We first conducted scRNA-seq analysis of individual sensory neurons from adult mouse DRG at 3 and 7 days after SNT. SNT was performed on one side of hind limbs so that DRG neurons on the contralateral side at the same level of the lumbar vertebrate bone served as an intact control. After enzymatically dissociating ipsilateral and contralateral L3–L5 DRGs, we manually picked similar number of small-, medium-, and large-diameter individual sensory neurons. We successfully constructed 106 scRNA-seq libraries (74 DRG neurons from axotomy side and 32 controls) (Fig. 1) that were quality-controlled by linear amplification of synthetic spike-in RNAs (Supplementary Fig. S1). At the depth of approximately one million mapped reads per library, each scRNA-seq library reveals ~7500 unique genes with the expression level at >10 TPM (Transcripts Per Kilobase Million) (Supplementary Fig. S2). As a direct control to the scRNA-seq, we also conducted RNA-seq with pooled neurons from control and injured DRGs at 3 days after SNT. Consistent with the previous microarray studies of entire DRGs^17^,^18^, our bulk RNA-seq samples showed known up-regulated RAGs and down-regulated voltage gated sodium channel genes (Supplementary Fig. S3, Supplementary Table S1). As expected, the data from single cells are equivalent to those using pooled DRG neurons (Supplementary Fig. S1a,b), further validating the high-quality of our scRNA-seq libraries.

### Unbiased characterization of sensory neuron subtypes after nerve transection

To gain insight into the intrinsic heterogeneity of sensory neurons in response to nerve injury, we first performed PCA of whole transcriptomes in both control and injury-side sensory neurons 3 days after SNT (Fig. 1a) and examined the expression levels of specific neuronal markers (Fig. 1b). We easily divided all the cells into two major clusters at the whole transcriptome level (Fig. 1). Cluster 1 includes all the injured neurons with high expression of many classic RAGs such as activating transcription factor 3 (*Atf3*) and c-Jun (*Jun*). By contrast, cluster 2 is composed of contra-lateral side control DRG neurons as well as a subset of ipsilateral sensory neurons (SNT side) with low expression of RAGs but high expression of the intrinsic regeneration inhibitor such as phosphatase and tensin homolog (*Pten*)^19^ (Fig. 1b, Supplementary Fig. S4f,g). The clustering of this subset of ipsilateral neurons with control neurons suggests that this subset of neurons may not be injured. Indeed, the axon branches of a small number of neurons in L3–L5 DRG do not contribute to sciatic nerve^20^, thus this subset of ipsilateral neurons may be spared by nerve injury in our model of SNT. Indeed, these cluster-2 non-responsive neurons have high expression of regeneration inhibitor PTEN and low expression of regeneration associated gene c-Jun on both protein and transcriptional level (Fig. 1c), distinctively different from injured *Jun*^+^ neurons in DRG at the ipsilateral SNT side^21^.

By examining the known neuronal markers, we categorized subtypes of DRG neurons from both injured (cluster 1) and intact (cluster 2) cells 3 days after SNT. For example, unmyelinated NP neurons have high expression of *P2rx3* but no expression of neurofilament heavy chain (*Nefh*). PEP neurons have expression of TRKA (*Ntrk1*) and *Calca* but no expression of Ret receptor (*Ret*). Proprioceptors have high expression of *Nefh* and parvalbumin (*Pvalb*). Low-threshold mechanoreceptors (LTMRs) co-express *Ntrk3, Nefh* and *Ret* mRNAs (Fig. 1f–i). In addition, we identified a small portion of *Nefh*^−^/tyrosine hydroxylase (*Th*)^+^ cells as C-fiber low-threshold mechanoreceptors (C-LTMRs) in cluster 2 based on the recent scRNA-seq subclassification^8^ (Fig. 1a,b, Supplementary Fig. S4b–g). We also correlated the soma size with the marker expression in each individual neuron. The overall size distribution of distinct DRG subtypes is in agreement with the recent report^9^, with large size for *Nefh*^+^ neurons and small size for *P2rx3*^+^ neurons (Supplementary Fig. S4a). Therefore, we successfully classified subtypes of injured neurons based on their specific markers, allowing us to further dissect the diversified injury responses in different subtypes of sensory neurons.

### Heterogeneous responses in subtypes of sensory neurons upon nerve injury

**Figure 2:**
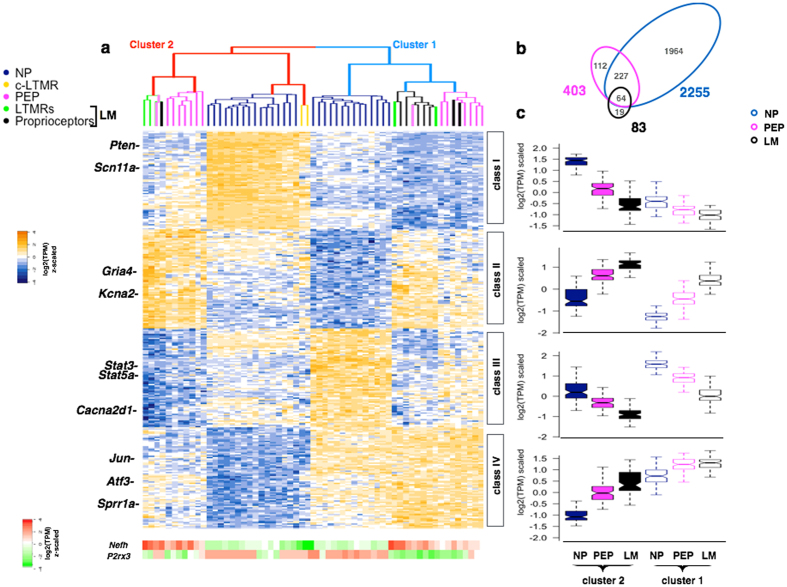
Heterogeneous responses of DRG subtypes after nerve injury. (**a**) Hierarchical clustering of genes with the highest loadings in the first and second principal components. Bottom: Scaled expression level of *Nefh* and *P2rx3* in each single-cell sample. (**b**) Venn diagram showing numbers of overlapped differentially regulated genes in three types of DRG neurons after injury. (**c**) Boxplot showing expression patterns of genes in class I–IV of cluster 1 and cluster 2 neurons for each subtype.

To understand the heterogeneous injury responses among subtypes of DRG neurons, we performed unsupervised hierarchical clustering using top 400 genes with the highest influence from the first and second principal components (Supplementary Table S2). This unbiased approach further partitions cluster 1 and 2 cells into three subgroups, namely LM, NP and PEP (Fig. 2a). As cluster 1 does not contain small-size C-LTMRs likely due to the limited sample cohort, we did not include C-LTMRs in the following analysis. In the six subgroups of control and injury neurons (LM, NP, and PEP neurons in cluster 1 and 2), we found distinct expression patterns for the following four classes of genes (Fig. 2c). Genes in Class I include voltage gated sodium channel gene Nav 1.9 (*Scn11a*) that is preferentially down-regulated in injured cluster I NP. PEP as well as LM (mainly proprioceptors) neurons, suggesting their relevance to the dysfunction of sensory neurons after injury^22^. Consistently, gene ontology analysis (geneontology.org) of the 100 class I genes shows the preferential enrichment (n = 18, enrichment >5-fold, p < 0.001) for biological process in regulation of metal ion transport (GO:0010959). Among the 101 Class II genes, we did not find statistically significant enrichment of GO terms. However, functionally relevant genes to sensory neurons such as glutamate receptor *Gria4* and voltage gated potassium channel *Kcna2* are exclusively down-regulated in injured NP neurons (Fig. 2a). The 100 genes in Class III are enriched for proteins intracellular protein transport (n = 12, enrichment >5-fold, p < 0.05; GO:0006886), including signal transducer and activator of transcription 5A (*Stat5a)* and nociception related gene voltage dependent calcium channel alpha 2/delta subunit 1 (*Cacna2d1)*, that are significantly up-regulated in injured cluster-1 NP and PEP neurons^23^. Finally, Class IV genes (n = 101) are usually up-regulated in all of the injury responsive cluster-1 neurons that include classic RAGs such as *Atf3* and c-*Jun*^24^ (Fig. 2a), suggesting that Class IV genes are related to nerve regeneration and functional recovery.

To gain further insight into differentially regulated genes in injured LM, NP and PEP neurons, we compared gene expression in these specific subtypes of neurons under control and injury conditions by using a Bayesian single-cell differential expression approach (SCDE)^25^. Interestingly, we found 2255 differentially regulated genes from NP neurons (1188 up-regulated, 1067 down-regulated) while only 403 differentially regulated genes in PEP neurons (222 up-regulated, 181 down-regulated) and 83 in LM neurons (51 up-regulated, 32 down-regulated) (P < 0.05) (Fig. 2b, Supplementary Table S3). Together, our data clearly showed differentially regulated transcriptional patterns in DRG neuronal subtypes, suggesting that the intrinsic heterogeneity of injury responses likely contributes to their differences in regeneration capacity and injury induced dysfunctions.

### Weighted gene co-expression network analysis (WGCNA) of differentially regulated injury response genes in DRG neurons

**Figure 3:**
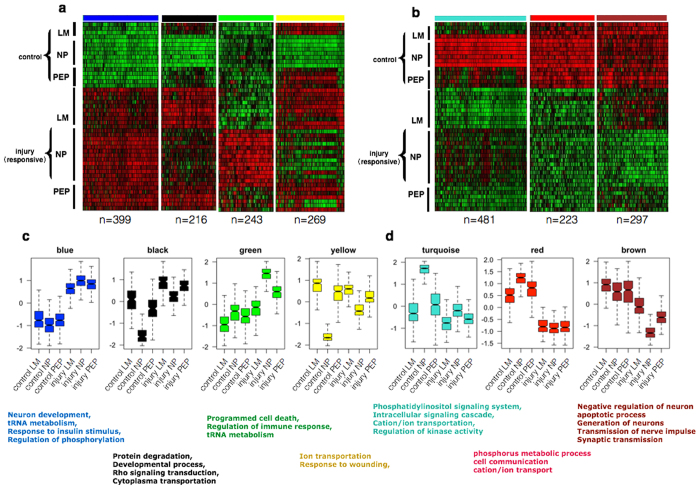
Gene correlation networks reveal differential regulation patterns of injury induced genes. (**a,b**) Heatmap showing relative expression of genes in seven gene modules identified by WGCNA of transcriptomes in control and injury-responsive neurons. (**c,d**) Boxplots showing expression patterns (scaled log_2_TPM) of injury induced genes in each WGCNA module. Representative GO terms of each significantly-regulated module are listed.

In small-size nociceptors, we expected to observe distinctive regulation of genes involved in neuronal cell death and neuropathic pain versus those related to nerve regeneration (such as Class IV genes described above in Fig. 2a). So we performed WGCNA to analyze a total of 2386 differentially regulated genes in both control and injured NP, PEP and LM neurons 3 days after SNT (Fig. 3, Supplementary Fig. S5a, Supplementary Table S4)^26^. Overall, we detected seven significant gene modules among all 2386 differentially regulated genes, with four mostly up-regulated genes modules (blue, black, green and yellow module) and three mostly down-regulated modules (brown, turquoise and red) after injury induction. As expected, each module has differentially regulation patterns among six neuronal subgroups (control and injured NP, PEP and LM neurons). For example, genes in blue modules (contain 399 genes) are co-regulated among all three DRG neuronal types before and after injury, including typical RAGs such as *Atf3*, c-*Jun,* SRY box-11 (*Sox11*) and small proline-rich protein 1A (*Sprr1a)*. GO analysis of blue module genes shows the enrichment in neuronal regeneration associated terms such as phosphorylation, tRNA metabolism and neuron development^18^. Genes in black module (216 genes) including heat shock 27 kDa protein 1 (*Hspb1)* and growth associated protein 43 (*Gap43*) are highly expressed in control and injured LM, which are up-regulated in both NP and PEP neurons after injury. GO of black module also shows the enrichment of neuron regeneration associated terms such as protein degradation and cytoplasmic transportation^27^. By contrast, genes in yellow module involve in calcium/sodium ion transport membrane and response to wounding, which are expressed at a lower level in the control NP neurons and up-regulated in a subset of NP neurons after injury. In particular, genes in green module (243 genes) are specifically up-regulated in NP neurons compare to LM and PEP neurons after injury (P < 4e–16), and are related to GO terms of cell death and immune responses, suggesting their function related to neuronal cell death in small unmyelinated nociceptors upon nerve injury. Genes in turquoise (481 genes) and red module (223 genes) are down-regulated after injury, involved in regulation of kinase/transferase activity and cation/ion transportation. Finally, 297 genes in brown module are expressed at similar level in control DRG subtypes, but specifically down-regulated in the injured NP neurons. These genes are functionally related to synapse assembly and organization, cell-cell signaling, and positive regulator of neural development (Fig. 3b,d). The down-regulation of these genes in NP neurons may be relevant to neuronal dysfunctions such as cell death and neuropathic pain after axotomy.

In order to determine the robustness of our classification, we cross-referred cluster genes in Class I-IV to the list of differentially regulated genes in seven WGCNA modules (Supplementary Fig. S5c). Significantly, we found that co-upregulated genes in black and blue modules, which are enriched for RAGs, including approximately 90% genes in Class IV, supporting the notion that Class IV genes contain both classic and novel RAGs. Similarly, approximately 40% of up-regulated Class III genes in injured NP neuron were found in green module. By contrast, the down-regulated genes in injured NP neurons identified from turquoise module overlaps with 90% of genes in Class I, which are functionally related to cation ion transport and indicative of neuronal dysfunction.

**Figure 4:**
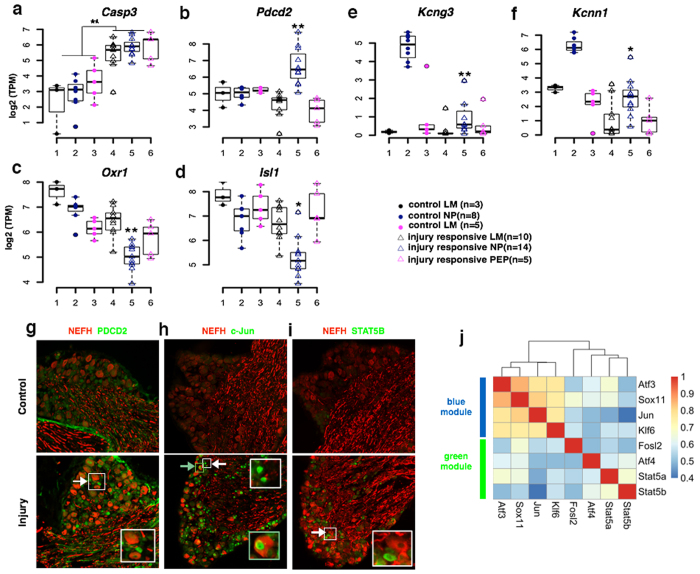
Correlation of transcriptional gene regulation with axotomy-induced cell death and neuropathic pain. (**a-d**) Boxplots showing the distribution of neuronal death/survival related gene expression of different DRG subtypes. *P < 0.05, **P < 0.01, unpaired t-test. (**e,f**) Boxplots showing the distribution of the gene expression of potassium channels in turquoise module among different DRG subtypes. (**g**) Immunofluorescence confirmed upregulation of apoptosis related PDCD2 in NEFH^−^ injured DRG neurons. PDCD2^high^/NEFH^−^ and PDCD2^low^/NEFH^+^ neurons (white arrow and white inset). (**h–i)** Immunofluorescence confirmed differential regulation of TFs in control and injured DRG neurons. NEFH^+^/c-Jun^high^ neuron (green arrow and green inset), NEFH^−^/c-Jun^high^ neuron (white arrow and white inset), NEFH^−^/STAT5b^high^ neuron (white arrow and white inset). (**j**) Correlation heatmap showing expression patterns of TFs in single DRG neurons.

In subtypes of sensory neurons after injury, we identified unique expression of cell survival and death related genes^28^. For example, we found that neuronal cell death gene *Caspase-3* in blue module is up-regulated in all three types of DRG neurons (Fig. 4a), whereas programmed cell death-2 (*Pdcd2*) in green module is significantly up-regulated only in injured NP neurons (Fig. 4b). Similarly, neuronal survival related genes ISL LIM homeobox 1 (*Isl1)* and oxidation resistance 1 (*Oxr1)* in brown module are preferentially down-regulated in injured NP neurons (Fig. 4c,d), consistent with their roles in neuronal cell loss^29^,^30^. The up-regulation of *Pdcd2* in small *Nefh* negative NP neurons was confirmed at the protein level via immunostaining (Fig. 4g). In addition, we also found that turquoise module comprises some voltage gated potassium channel genes such as *Kcng3* and *Kcnn1* that are specifically down-regulated in injured NP neurons (Fig. 4e,f), potentially associated with pain hypersensitivity in injured small cutaneous NP neurons^31^.

Many genes in blue and black modules are co-upregulated in all three types of injured sensory neurons related to regeneration function in protein phosphorylation, translation, neuron development, protein degradation and cytoplasm transportation. Indeed, we observed a significant overlap of the blue (31.6%) and black (20.0%) module genes with injury-response genes in DRGs reported by a recent study (Supplementary Table S5)^32^. We further cross-referred up-regulated genes from blue, black, green and yellow modules with known RAGs discovered from other studies (Supplementary references 1–43). Remarkably, we found 26 RAGs in blue module, 9 in black module, 2 in yellow module and 1 in green module (Supplementary Table S6), consistent with the preferential enrichment of putative RAGs in blue and black module compared to other modules. Since RAGs include transcription factors (TFs) that play important regulatory roles during neuron regeneration and degeneration^33^, we then characterized the injury associated TFs in our functional gene modules. We found that a group of TFs in blue module clustered closely together with many known RAGs such as *Atf3, c-Jun, Sox11* and kruppel-like factor 6 (*Klf6*), suggesting their co-regulation relationships during axon regeneration. Similarly, the TFs in green module such as *Atf4* and *Stat5a/b* did not group with known RAGs, suggesting their functions in injured neurons may not be related to nerve regeneration and functional recovery (Fig. 4j). We confirmed the differential regulation of c-Jun and STAT5B in blue and green modules at protein level, respectively (Fig. 4h,i).

### Distinct molecular program of LM neurons at multiple time points of SNT

**Figure 5:**
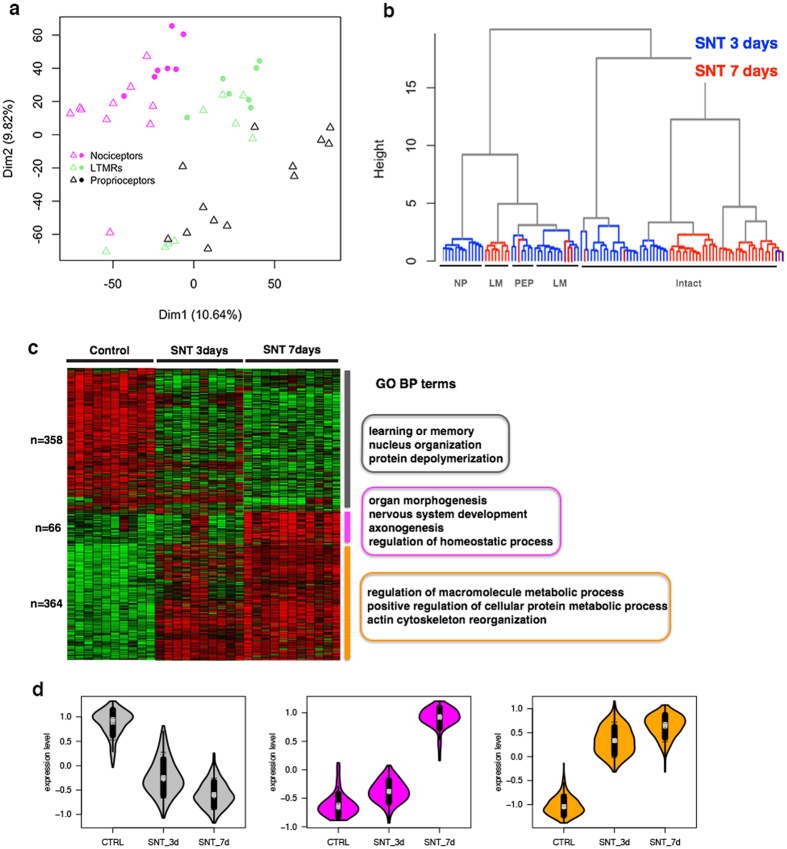
Conserved injury responses in LM-subtype neurons at different time points of SNT. (**a**) PCA analysis of mRNA transcriptome in LM neuron subtypes at 7 days after SNT respectively. Triangles represent DRG neurons collected on the SNT side, points represent DRG neurons on the control side. Individual DRG neurons were annotated according to their different expression patterns of marker genes as shown in Supplementary Fig. S6. (**b**) Hierarchical clustering of transcriptome profiles revealed closer cell-cell distance between day-3 and day-7 after SNT in LM neurons compared to other neuronal subtypes. (**c**) Heatmap showing relative expression of differentially expressed genes which are divided into 3 representative modules by WGCNA. Gene ontology (BP) analysis of each individual module is shown on the right. (**d**) Violin plot showing dynamic gene expression of gray, magenta and orange modules at different time points after SNT.

As stated above, we observed dramatic heterogeneous injury responses among NP, LM and PEP neurons days after SNT. We then asked whether the molecular program in response to injury will sustain at a longer time period. Therefore, we analyzed the transcriptomes of DRG neurons 7 days after SNT. After examining the expression patterns of specific markers, we found that the majority of *Atf3*^+^ injured neurons are LM neurons such as LTMRs (*Ntrk3*^+^/*Pvalb*^*−*^) and Proprioceptors (*Ntrk2*^+^/*Pvalb*^+^*, Ntrk3*^+^/*Pvalb*^+^) (Fig. 5a, Supplementary Fig. S6a–f). Hierarchical clustering analysis of transcriptome profile showed that day-7 cluster closer to day-3 LM neurons than other injured neuronal subtypes (Fig. 5b). We detected 771 differentially regulated genes (420 up-regulated and 351 down-regulated) on day 7 when compared to 87 from day 3 LM neurons. In order to comprehensively analyze the injury responses of LM neurons at different time points, we categorized the differentially regulated genes from day-3 and day-7 LM neurons into 3 modules according to their co-expression patterns (Fig. 5c,d, Supplementary Table S4). Briefly, gray module contains 358 gradually down-regulated genes, which are associated with learning and memory, nucleus organization and protein depolymerization. Magenta module has 66 up-regulated genes in a small number of day-3 SNT neurons and all the day-7 SNT neurons, while orange module has 364 genes gradually up-regulated at different time points of SNT. The magenta and orange modules are associated with nerve regeneration such as organ morphogenesis, nerve system development and axonogenesis. Together, these results demonstrated that LM neurons display dynamic injury-induced gene expression with a relatively conserved regeneration related gene co-expression network both 3 and 7 days after SNT.

To determine whether the transcriptional changes in LM neurons at day-7 after SNT exhibit distinct patterns compared to other neuronal subtypes such as NP neurons, we cross-referenced the three modules in LM neurons on day 7 to the seven co-expression modules differentially regulated in LM, PEP and NP neurons on day 3. Direct module comparison identified significant overlap of gene modules between day 3 and day 7. For instance, up-regulated genes in orange module are significantly overlapped with co-regulated modules such as blue module (156 overlapped genes, P < 2.64e–41, hypergeometric test) and black module (40 overlapped genes, P < 0.015), whereas down-regulated genes in gray modules are significantly overlapped with co-regulated red module (52 overlapped genes, P < 3.82e–5). We did not observe significant overlap of injury induced genes in day-7 LM neurons with those of green, yellow and turquoise modules specifically regulated in NP neurons. Although our results were still preliminary, it suggests that each DRG neuronal subtypes may maintain a distinguished transcriptional program during a longer time course of nerve injury/recovery.

## Discussion

By single-cell RNA-seq analysis of mRNA transcriptome of injured DRG neurons, we, for the first time, observed genome-wide heterogeneous injury responses among various subtypes of DRG neurons. Using weighted gene network analysis, we identified injury-responsive genes related to abnormal pain and cell death particularly in small-size nociceptors as well as shared gene modules related to regeneration that are co-regulated among all three subtypes of sensory neurons. Consistent with the unique transcriptional change in a specific subtype of sensory neurons, we also uncovered the robust transcriptional alterations in the pathways of cell death and neuropathic pain in injured small unmyelinated neurons.

The poor functional recovery after peripheral nerve injury is often associated with delayed loss of cutaneous neurons, in which apoptosis is induced by alterations of electrical activity and loss of target derived neurotrophic factor support^34^. It is known that dynamic expression of pro-apoptotic mediator *Bax,* pro-survival factor *Bcl-2* and *Caspase-3* is correlated to injury induced neuronal death^35^. While NP neurons are more prone to cell death, we found that *Caspase-3* is up-regulated in all the injured neuronal subtypes (Fig. 4a). It has been reported that sensory neurons can survive for a long time with high expression of *Caspase-3* in a diabetes rat model^36^. We therefore suggest that other factors in addition to Caspase-3 are involved in the induction of neuronal cell death in post-traumatic cutaneous sensory neurons such as NP neurons. In fact, we observed the upregulation of *Pdcd2* gene and down-regulation of neuron survival factor *Isl1* and *Oxr1* specifically in injured NP neurons, consistent with our suggestion^29^,^37^.

Peripheral neuropathic pain is caused by malfunction of receptors, enzymes, and voltage-dependent ion channels in DRG neurons, as well as changes in the nociceptive pathway at synapses in the central nervous system^31^,^38^. Voltage gated ion channels are drug targets for treating neuropathic pain due to their association with generation and conduction of pain hypersensitivity^39^. For example, gain-of-function and up-regulation of voltage gated sodium channel Nav1.7–1.9 (*Scn9a, Scn10a, Scn11a*) are related to inflammation induced pain^40^ while down-regulation of potassium channels are related to hyperexcitable phenotype^41^,^42^. Consistent with the previous findings^17^, we found the majority of genes encoding voltage gated sodium channels such as Nav1.7–1.9 are down-regulated in our axotomy model (Supplementary Fig. 3b). Nevertheless, we found specifically down-regulation of turquoise module genes *Kcng3* and *Kcnn1* in NP neurons. Because down-regulation of potassium channel genes are implicated in pain hypersensitivity^22^,^43^,^44^, our results suggested that these *Kcng3* and *Kcnn1* in our axotomy model are the novel target genes contributing to pain hypersensitivity as well.

Although previous genome-wide screens have yielded numerous candidate injury-induced genes, only a small proportion of these genes are RAGs^4^. In this study, we were able to find more candidate RAGs from shared up-regulated genes in injured NP, PEP and LM neurons (e.g. Class IV genes in Fig. 2 and Supplementary Table 2 and candidate genes in blue and black modules via WGCNA). By cross-comparing our gene list with up-regulated genes in a model of pre-conditioned DRG^32^ as well as known RAGs (Supplementary Table S6), we uncover additional novel RAGs in our injury model via scRNA-seq. These novel RAG genes are connected by the known RAGs genes such as *Atf3, c-Jun, Sox11* and *Klf6* in regeneration gene network analysis as well^45^,^46^,^47^,^48^,^49^.

In summary, our study not only reveals tremendous heterogeneity of sensory neurons in response to injury, but also identifies novel target genes associated with either neuronal cell death and physiological pain or nerve regeneration and neuronal recovery.

## Methods

### Experimental animals and tissue processing

The experiment was performed on two month old adult CAST/Eij ♂ cross C57BL/6♀ F1 female mice and C57BL/6 mice of both gender. The animal experiments were carried out in accordance with the protocols approved by Animal Experiment Administration Committee of Tongji University School of Medicine, China (No. TJLAC-015-022). Animals were kept in cages in groups, with sufficient food and water, under 12-h light-dark cycle conditions. All surgical procedures were performed under general anesthesia with intraperitoneal injections using a mixture of ketamine (100 mg/kg) and xylazine (10 mg/kg). For the sciatic nerve transection (axotomy), the right side sciatic nerve was exposed at the mid thigh level and sectioned distally. The wound was sutured in two layers, and the animals were allowed to recover. At 3 days and 7 days post surgery, animals were performed euthanasia by CO_2_ and decapitation, L3–L5 DRG from mice of both ipsilateral and contralateral side were dissected and dissociated into single cells. Single DRG soma was manually picked in the lowest possible volume (preferably ≤0.5 μl, possibly 0.3 μl) using a micro capillary pipette into a 0.2-ml thin-wall PCR tube contains 4 μl smart-seq2 lysis buffer. Cell sizes were measured and recorded during picking. DRG neuron were randomly picked with almost equal number of small, medium and large diameter neurons in order to incorporate most subtypes. Except for the enzyme dissociation, all the dissection procedure were performed on ice in order to reduce RNA degradation.

### Single cell RNA-seq and reads processing for differentially gene expression analysis

Whole transcriptome amplification in tubes were performed following Smart-seq2 protocol with minor edition^50^. Briefly, the cells were lysed and poly-A RNA was reverse transcribed by superscript III reverse transcription enzyme using a template switch fashion. cDNA were then amplified by KAPA polymerase for 18 PCR cycles. After purification, 0.1 ng cDNA were used for Nextera tagmentation and library construction. ERCC RNA spike-in Mix (Ambion, Life Technologies) was added to the lysis reaction and processed in parallel with poly-A RNA. Libraries were sequenced on illumina Hi-seq2000 and Next-seq500 pipeline. CASAVA 1.8.2 was used to separate out the data for each single cell by using unique barcode combinations from the Nextera XT preparation and to generate fastq files. Raw reads past quality control were mapped by bowtie2 using default parameter, gene expression level was quantified as transcripts per million reads (TPM) using RSEM to known exons using Refseq gene annotation downloaded from UCSC genome browser. Ribosomal RNA annotations were removed from reference file before gene expression quantification.

We performed smart-seq2 single cell RNA-seq on 106 L5 single DRG neuron, including 74 single neurons from SNT side and 32 cells from their contralateral control side. After filtering out genes mapped on ribosomal RNA, each of the single cell samples generated an average of 0.835 ± 0.397 (average ± s.d) million unique mapped reads on genes and detected 7569 ±1456 (average ±s.d) genes with TPM expression level larger than 10. For each side, a total of 50 dissociated DRG neurons with almost equal number of small, medium and large diameter neurons were lysed in one tube and split into three parts in order to construct cell population control. To ensure the fidelity of the data, we checked the correlation of spike-in RNA controls. The levels of RNA spikes determined by RNA sequencing correlated strongly with each other with minimal variances (Supplementary Fig. 1).

### Principal component analysis (PCA) and Hierarchical Clustering Analysis

Before the PCA analysis, we determined a cut-off for genes that had greater than 10 TPM in at least 5 samples. We then did a quantile normalization on the remaining data followed by log transform, leaving 12145 genes for further analysis. PCA was performed using an R package FactoMineR with scale.unit = TRUE normalization. PCA plot cluster all the samples into two groups, we excluded 11 samples in the distal cluster as outliers, leaving the remaining 106 single DRG samples clustering with 6 bulk sample control. We used the first two principal components coefficient scores to determine genes which highest magnitude in subclassifying DRG samples. In Fig. 2a, a total of 400 genes with highest magnitude in PC1 and PC2 were used for hierarchical clustering analysis. Gene expression in each sample was scale normalized and clustered based on their spearman correlation. The separation was robust and repeatable using either 400 genes or all 12145 genes.

### Differentially gene expression analysis

An R package DEseq was used to calculate differentially expression genes between bulk samples. We determined significantly differentially expressed genes with the criteria of mean expression level fold change >2 and FDR adjusted P-value < 0.05. To identify differentially expressed genes in individual neuronal groups, we used the recently published Bayesian approach to single-cell differential expression (SCDE) analysis method^25^ to compare each cell class against all other clusters. Briefly, raw count data from each group were fit a SCDE error model in order to estimate both the likelihood of a gene being expressed at any given average level in each subpopulation as well as estimating the probability of a dropout event for each gene and in each cell simultaneously. Differentially gene expression analysis were then calculated using the joint posterior probability of expression in each cell group. Finally, genes with fold expression difference posterior larger than 2 and with FDR adjusted P-value < 0.05 were identified as differentially expressed genes. We used unparied t-test and one-way ANOVA to test differentially expression of individual interested genes in three types of DRG neuron.

### Immunohistochemistry

Male adult mice were euthanized by CO_2_ and decapitated, DRGs were dissected out and postfixed in 4% PFA for 1 h at 4 °C. Samples were subsequently washed in PBS and cryopreserved at 4 °C overnight with 30% sucrose in PBS. Tissue was then embedded in OCT and frozen at −20 °C. Samples with 10 μm sections were cut on a freezing sledge microtome (Leica Microsytems) and air dry in room temperature for two hours to prepare for staining. Two mice were used for each immunostaining, showing similar results. Sections were permeabilized in PBS+0.5%Triton-x100 for 10 min. Following blocking in 1%BSA PBS for 1 hour, samples were then incubated in primary antibody, including chicken anti-NEFH (abcam, ab4680), rabbit anti-PTEN (abcam, ab32199), rabbit anti-c-JUN(abcam, ab31419), rabbit anti-STAT5B (abcam, ab76319), rabbit anti-PDCD2 (Proteintech,10725-1-AP) at room temperature for 2 hours. For detection, Donkey 488- and Cy3-conjugated Alexa secondary against rabbit and goat were used at dilution of 1:400. (Jackson ImmunoResearch 705-545-003 and 703-166-155) for one hour in the room temperature. After PBS washes, slides were subsequently mounted with mounting medium (darko) and visualized with a Nikon Fluorescent microscope.

### Weighted gene co-expression network analysis

To perform WGCNA, a matrix of signed Pearson correlation between all pairs of transcripts was computed. This correlation matrix was raised to power β = 14 to calculate a adjacency matrix. To minimize the noise and spurious associations, the adjacency matrix was transformed to topological overlap matrix (TOM). The matrix 1-TOM was used as the input of average linkage hierarchical cluster, and genes with similar expression pattern were clustered together. The expression profile of a given module was represented by its first principal component (as known as module eigengene, ME) which can explain the most variation of the module expression levels. Module membership (also known as module eigengene based connectivity, kME) of each genes was calculated by correlating the gene expression profile with ME.

### Function analysis

Gene ontology enrichment analysis was carried out by combined analysis of Bioconductor package “topGO” [PMID:16606683] and online DAVID GO database: https://david.ncifcrf.gov/tools.jsp. Terms were accepted if they are hitted more than 1 gene and Fisher’s Exact Test P-value < 0.05.

## Additional Information

**How to cite this article**: Hu, G. *et al*. Single-cell RNA-seq reveals distinct injury responses in different types of DRG sensory neurons. *Sci. Rep.* **6**, 31851; doi: 10.1038/srep31851 (2016).

**Accession code**: Gene Expression Omnibus: raw data for individual libraries and normalized data for all sequenced library, GSE71453.

## Supplementary Material

Supplementary Information

Supplementary Table 1–5
