# Supplementary material for: Single-cell RNA-seq reveals distinct injury responses in different types of DRG sensory neurons
Source: Sci Rep. 2016 Aug 25;6:31851. doi: 10.1038/srep31851 (PMC4997251; doi:10.1038/srep31851)
Supplement: Supplementary Information [file srep31851-s1.doc]

**Supplementary information to:**

Single-cell RNA-seq reveals distinct injury responses in different types of DRG sensory neurons

Ganlu Hu^1,2,3^, Kevin Huang^2^, Youjin Hu^2,3^, Guizhen Du^2^, Zhigang Xue^3^, Xianmin Zhu^1,4*^, Guoping Fan^1, 2, 5*^

^1^School of Life Sciences and Technology, Tongji University, Shanghai 200092, China;

^2^Department of Human Genetics, David Geffen School of Medicine, University of California Los Angeles, Los Angeles CA 90095, USA;

^3^Translational Center for Stem Cell Research, Tongji Hospital, Department of Regenerative Medicine, Tongji University School of Medicine, Shanghai 20065,China

^4^Shanghai Pulmonary Hospital, Tongji University School of Medicine, Shanghai 200433, China

^5^Wuxi Medical School, Jiangnan University, Wuxi City, Jiangsu Province, China


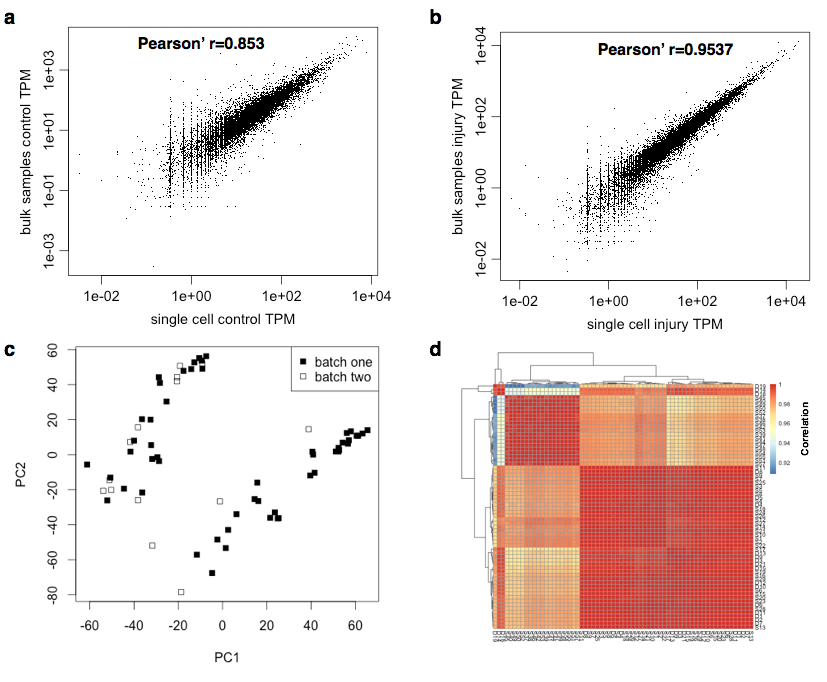


**Figure S1:** **Assessment of technical and biological variation, batch effect and reproducibility of scRNA-seq**

(**a-b**) The correlation of the transcript levels of all genes in the single cells with those in the pooled DRG samples (Pearson correlation coefficients r are given);

(**c**) PCA analysis of single-cell DRG samples from two batches of the experiments;

(**d**) The correlation of transcript levels (TPM) of 92 ERCC RNA spike-ins in each single-cell DRG sample.


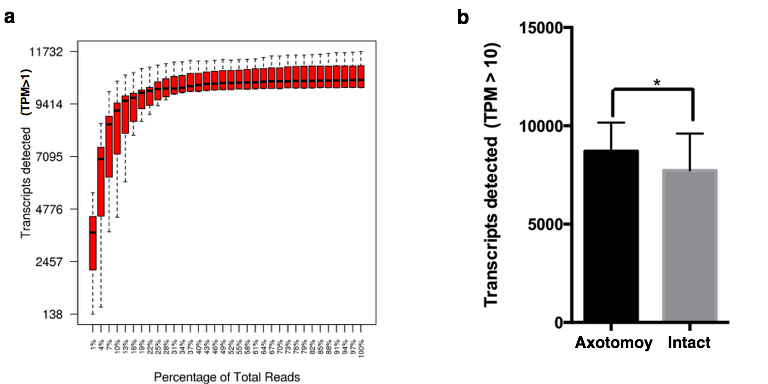


**Figure S2:** **Transcript number detection in scRNA-seq samples.**

**(a)** Saturation analysis revealed that the sequencing depth is enough for the detection of most genes expressed in single cells.

(**b**) Bar plot compared the transcript numbers detected in between SNT treated single DRG neuron and intact single DRG neuron. Mean ± s.e.m. *P < 0.05. unequal variances t-test.


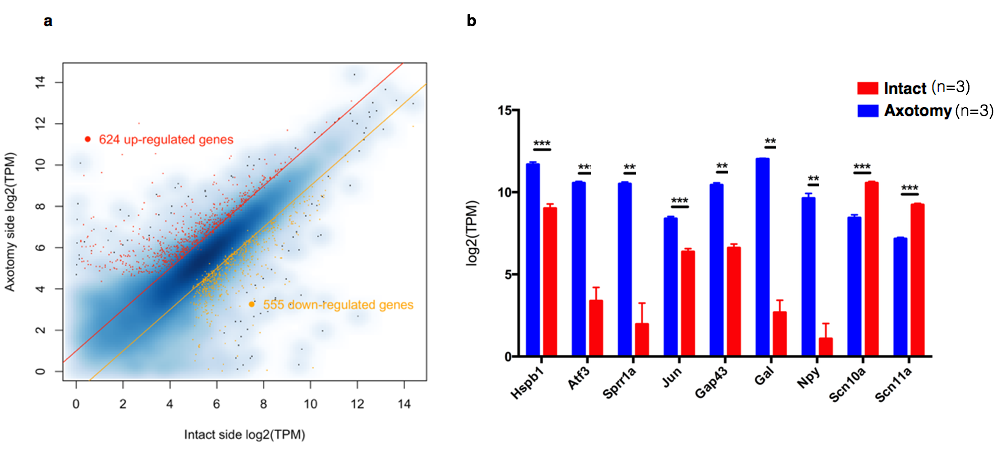


**Figure S3: Identification of the genes induced by SNT using bulk samples.**

**(a)** Gene expression analysis of bulk L5 DRG neurons in response to SNT along with their contralateral control side. The significant up-regulated genes are marked red, and the downregulated genes are marked blue. (Fold change of average expression level >2, FDR adjusted P <0.05)

(**b**) Comparison of representative differentially regulated genes between the injury and control neurons. Mean ± s.e.m. **P < 0.01, ***P < 0.001, unpaired t-test.

**
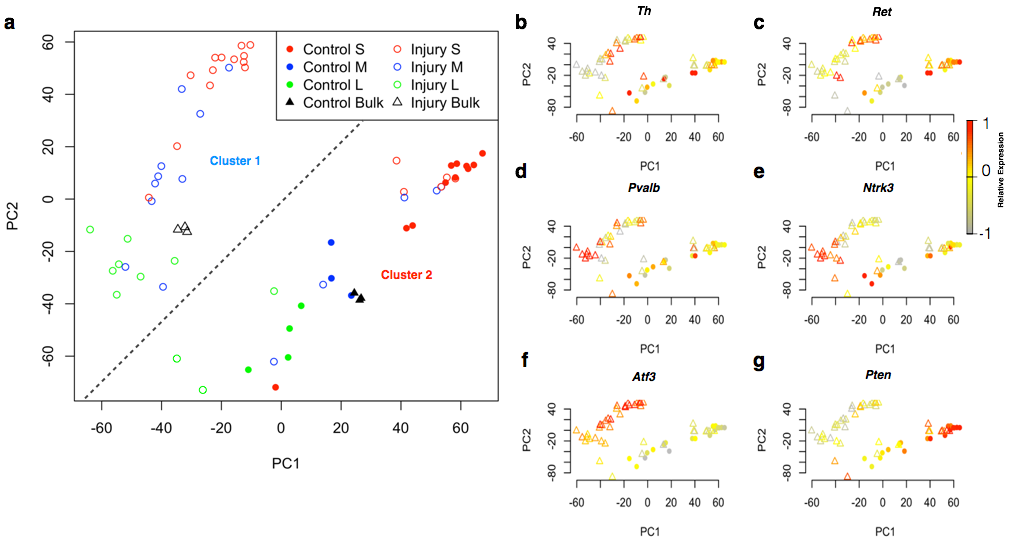
**

**Figure S4: PCA analysis of single sensory neuron subtypes with differential injury responses after SNT**.

**(a)** Unbiased PCA analysis of mRNA transcriptome in individual sensory neurons. Different cell-body diameters of both control and injured DRG neurons were mapped back to each sample.

(**b-g**) Relative expression levels of DRG neuron type-specific markers and RAGs were mapped back to each sample. Color key represents normalized gene expression with the highest expression marked red and the lowest marked gray.


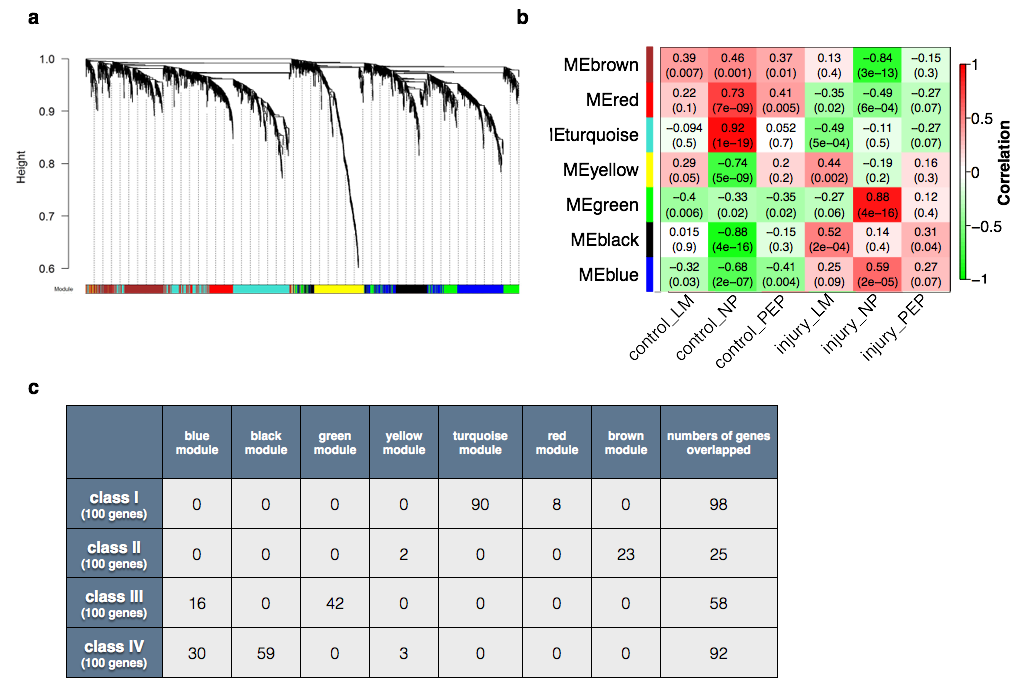
**Figure S5: Network analysis of co-expressed gene regulation in three subtypes of sensory neurons in response to SNT injury.**

**(a)** Hierarchical cluster tree showing co-expressed gene modules identified by WGCNA, modules correspond to each branches are labeled by colors.

**(b)** Heatmap showing the significance of each gene module regulated in six DRG subtypes. Each cell contains Pearson correlations of gene expression profile from each subtype with each module’s ME (the first principal component) and their corresponding P values.

**(c)** Table showing the numbers of class I-IV genes overlapped with genes from 7 WGCNA modules.


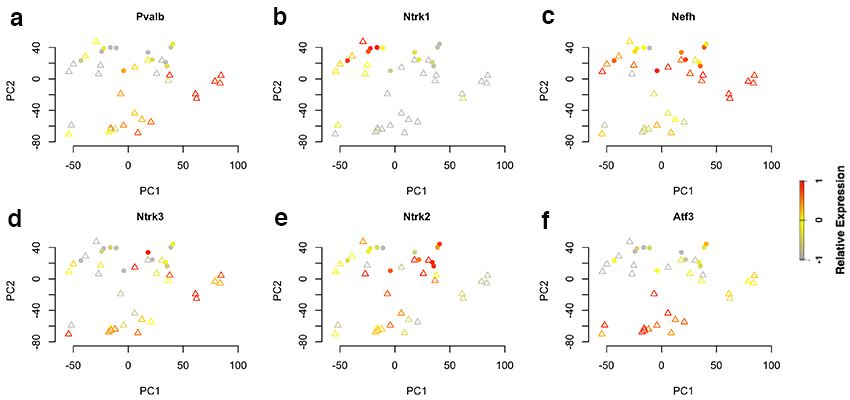


**Figure S6 Single-cell transcriptome analysis of sensory neuron subtypes at 7 days after SNT.**

**(a-f)** Unbiased PCA analysis of mRNA transcriptome in individual sensory neurons, with relative expression level of DRG neuron type-specific marker mapped back to each sample. Color key represents normalized gene expression with the highest expression marked red and the lowest marked gray.

**Supplementary table S6. Known RAGs in blue, black, green and yellow modules.**

——————————————————————————————————————

Gene Symbol Module Citation Gene Function

(relate to injury responses)

——————————————————————————————————————

*Atf3* blue ^1,2^ promote nerve regeneration

*Itga7* blue ^3^ promote neurite outgrowth

*Stmn4* blue ^4^ promote microtubule assemble

*Flrt3* blue ^5^ promotes neurite outgrowth

*Tubb6* blue ^6^ microtubule assemble

*Hn1* blue ^7^ nerve regeneration

*Bach1* blue ^8^ axon growth inhibitor

*Cttn* blue ^9^ promotes neurite outgrowth

*Sema6a* blue ^10^ axon growth inhibitor

*Sprr1a* blue ^11^ promotes neurite outgrowth

*Sox11* blue ^12^ accelerate in vivo nerve regeneration

*Camk1*  blue ^13^ promote peripheral nerve regeneration

*Gadd45a* blue ^14^ anti-apoptosis

*Cdkn1a* blue ^15^ enhance axonal regeneration

*Itgb1* blue ^16^ promote microtubule assemble

*Myo10* blue ^17^ induce filopodia formation

*Smad1* blue ^18^ promote neurite outgrowth

*Rgs12* blue ^19^ required for neurite outgrowth in PC12 cells

*Fgf3* blue ^20^ induce neuron apoptosis

*Klf6* blue ^21^ promote neurite outgrowth

*Tuba1c* blue ^22^ promote axonal regeneration

*Nfil3* blue ^23^ axon growth inhibitor

*Jun* blue ^24^ promote axonal regeneration

*Ndel1* blue ^25^ promote axonal regeneration

*Hdac6*  blue ^26^ axon growth inhibitor

*Rtn4* blue ^27^ axon growth inhibitor

*Gal* blue ^28^ promote axonal regeneration

*Gadd45b* blue ^29^ promote axonal regeneration

*Ptpn1*  blue ^30^ promote neurite outgrowth

*Serpinf1* blue ^31^ neuron-protection effect

*Hdac3*  black  ^32^ induce neuron apoptosis

*Lancl1* black ^33^ anti-apoptosis

*Gap43*  black ^34^ promote microtubule assemble

*Hspb1* black  ^35^ anti-apoptosis

*Csrp1*  black  ^36^ promote axonal regeneration

*Adcyap1* black ^37^  promote axonal regeneration

*Arc* black ^30^ Stimulates extension in neuroblastoma

*Cfl* black ^38^ promote neurite outgrowth

*Rora* black ^39^ anti-apoptosis

*Ncs1*  black ^40^ promote axonal regeneration

*Skil* green ^41^ promote axonal regeneration

*Rara* yellow ^42^ promote axonal regeneration

*Cntf* yellow ^43^ promote axonal regeneration

——————————————————————————————————————

# Reference

1. Reid, A.J., Welin, D., Wiberg, M., Terenghi, G. & Novikov, L.N. Peripherin and ATF3 genes are differentially regulated in regenerating and non-regenerating primary sensory neurons. Brain research 1310, 1-7 (2010).

2. Seijffers, R., Mills, C.D. & Woolf, C.J. ATF3 increases the intrinsic growth state of DRG neurons to enhance peripheral nerve regeneration. The Journal of neuroscience : the official journal of the Society for Neuroscience 27, 7911-7920 (2007).

3. Werner, A. et al. Impaired axonal regeneration in alpha7 integrin-deficient mice. The Journal of neuroscience : the official journal of the Society for Neuroscience 20, 1822-1830 (2000).

4. Iwata, T. et al. Increased expression of mRNAs for microtubule disassembly molecules during nerve regeneration. Brain research. Molecular brain research 102, 105-109 (2002).

5. Tsuji, L. et al. FLRT3, a cell surface molecule containing LRR repeats and a FNIII domain, promotes neurite outgrowth. Biochemical and biophysical research communications 313, 1086-1091 (2004).

6. Reddien, P.W., Bermange, A.L., Murfitt, K.J., Jennings, J.R. & Sanchez Alvarado, A. Identification of genes needed for regeneration, stem cell function, and tissue homeostasis by systematic gene perturbation in planaria. Developmental cell 8, 635-649 (2005).

7. Zujovic, V. et al. The facial motor nucleus transcriptional program in response to peripheral nerve injury identifies Hn1 as a regeneration-associated gene. Journal of neuroscience research 82, 581-591 (2005).

8. Kanno, H. et al. Genetic ablation of transcription repressor Bach1 reduces neural tissue damage and improves locomotor function after spinal cord injury in mice. Journal of neurotrauma 26, 31-39 (2009).

9. Spillane, M. et al. Nerve growth factor-induced formation of axonal filopodia and collateral branches involves the intra-axonal synthesis of regulators of the actin-nucleating Arp2/3 complex. The Journal of neuroscience : the official journal of the Society for Neuroscience 32, 17671-17689 (2012).

10. Shim, S.O. et al. PlexinA2 limits recovery from corticospinal axotomy by mediating oligodendrocyte-derived Sema6A growth inhibition. Molecular and cellular neurosciences 50, 193-200 (2012).

11. Bonilla, I.E., Tanabe, K. & Strittmatter, S.M. Small proline-rich repeat protein 1A is expressed by axotomized neurons and promotes axonal outgrowth. The Journal of neuroscience : the official journal of the Society for Neuroscience 22, 1303-1315 (2002).

12. Jing, X., Wang, T., Huang, S., Glorioso, J.C. & Albers, K.M. The transcription factor Sox11 promotes nerve regeneration through activation of the regeneration-associated gene Sprr1a. Experimental neurology 233, 221-232 (2012).

13. Elziere, L. et al. CaMKK-CaMK1a, a new post-traumatic signalling pathway induced in mouse somatosensory neurons. PloS one 9, e97736 (2014).

14. Lin, C.R. et al. GADD45A protects against cell death in dorsal root ganglion neurons following peripheral nerve injury. Journal of neuroscience research 89, 689-699 (2011).

15. Tanaka, H. et al. Cytoplasmic p21(Cip1/WAF1) enhances axonal regeneration and functional recovery after spinal cord injury in rats. Neuroscience 127, 155-164 (2004).

16. Lei, W.L. et al. Laminin/beta1 integrin signal triggers axon formation by promoting microtubule assembly and stabilization. Cell research 22, 954-972 (2012).

17. Bennett, R.D., Mauer, A.S. & Strehler, E.E. Calmodulin-like protein increases filopodia-dependent cell motility via up-regulation of myosin-10. The Journal of biological chemistry 282, 3205-3212 (2007).

18. Zou, H., Ho, C., Wong, K. & Tessier-Lavigne, M. Axotomy-induced Smad1 activation promotes axonal growth in adult sensory neurons. The Journal of neuroscience : the official journal of the Society for Neuroscience 29, 7116-7123 (2009).

19. Willard, M.D. et al. Selective role for RGS12 as a Ras/Raf/MEK scaffold in nerve growth factor-mediated differentiation. The EMBO journal 26, 2029-2040 (2007).

20. Jungnickel, J., Gransalke, K., Timmer, M. & Grothe, C. Fibroblast growth factor receptor 3 signaling regulates injury-related effects in the peripheral nervous system. Molecular and cellular neurosciences 25, 21-29 (2004).

21. Veldman, M.B., Bemben, M.A., Thompson, R.C. & Goldman, D. Gene expression analysis of zebrafish retinal ganglion cells during optic nerve regeneration identifies KLF6a and KLF7a as important regulators of axon regeneration. Developmental biology 312, 596-612 (2007).

22. Veldman, M.B., Bemben, M.A. & Goldman, D. Tuba1a gene expression is regulated by KLF6/7 and is necessary for CNS development and regeneration in zebrafish. Molecular and cellular neurosciences 43, 370-383 (2010).

23. MacGillavry, H.D. et al. NFIL3 and cAMP response element-binding protein form a transcriptional feedforward loop that controls neuronal regeneration-associated gene expression. The Journal of neuroscience : the official journal of the Society for Neuroscience 29, 15542-15550 (2009).

24. Besirli, C.G. & Johnson, E.M., Jr. JNK-independent activation of c-Jun during neuronal apoptosis induced by multiple DNA-damaging agents. The Journal of biological chemistry 278, 22357-22366 (2003).

25. Toth, C. et al. Ndel1 promotes axon regeneration via intermediate filaments. PloS one 3, e2014 (2008).

26. Rivieccio, M.A. et al. HDAC6 is a target for protection and regeneration following injury in the nervous system. Proceedings of the National Academy of Sciences of the United States of America 106, 19599-19604 (2009).

27. Watari, A. & Yutsudo, M. Multi-functional gene ASY/Nogo/RTN-X/RTN4: apoptosis, tumor suppression, and inhibition of neuronal regeneration. Apoptosis : an international journal on programmed cell death 8, 5-9 (2003).

28. Horie, H. et al. Oxidized galectin-1 stimulates macrophages to promote axonal regeneration in peripheral nerves after axotomy. The Journal of neuroscience : the official journal of the Society for Neuroscience 24, 1873-1880 (2004).

29. Liu, B. et al. Gadd45b Mediates Axonal Plasticity and Subsequent Functional Recovery After Experimental Stroke in Rats. Molecular neurobiology 52, 1245-1256 (2015).

30. Donai, H. et al. Interaction of Arc with CaM kinase II and stimulation of neurite extension by Arc in neuroblastoma cells expressing CaM kinase II. Neuroscience research 47, 399-408 (2003).

31. Tombran-Tink, J., Chader, G.G. & Johnson, L.V. PEDF: a pigment epithelium-derived factor with potent neuronal differentiative activity. Experimental eye research 53, 411-414 (1991).

32. Schmitt, H.M., Pelzel, H.R., Schlamp, C.L. & Nickells, R.W. Histone deacetylase 3 (HDAC3) plays an important role in retinal ganglion cell death after acute optic nerve injury. Molecular neurodegeneration 9, 39 (2014).

33. Huang, C. et al. Developmental and activity-dependent expression of LanCL1 confers antioxidant activity required for neuronal survival. Developmental cell 30, 479-487 (2014).

34. Schreyer, D.J. & Skene, J.H. Fate of GAP-43 in ascending spinal axons of DRG neurons after peripheral nerve injury: delayed accumulation and correlation with regenerative potential. The Journal of neuroscience : the official journal of the Society for Neuroscience 11, 3738-3751 (1991).

35. Ma, C.H. et al. Accelerating axonal growth promotes motor recovery after peripheral nerve injury in mice. The Journal of clinical investigation 121, 4332-4347 (2011).

36. Ma, L., Yu, Y.M., Guo, Y., Hart, R.P. & Schachner, M. Cysteine- and glycine-rich protein 1a is involved in spinal cord regeneration in adult zebrafish. The European journal of neuroscience 35, 353-365 (2012).

37. Armstrong, B.D. et al. Impaired nerve regeneration and enhanced neuroinflammatory response in mice lacking pituitary adenylyl cyclase activating peptide. Neuroscience 151, 63-73 (2008).

38. Meberg, P.J., Ono, S., Minamide, L.S., Takahashi, M. & Bamburg, J.R. Actin depolymerizing factor and cofilin phosphorylation dynamics: response to signals that regulate neurite extension. Cell motility and the cytoskeleton 39, 172-190 (1998).

39. Boukhtouche, F. et al. Human retinoic acid receptor-related orphan receptor alpha1 overexpression protects neurones against oxidative stress-induced apoptosis. Journal of neurochemistry 96, 1778-1789 (2006).

40. Yip, P.K., Wong, L.F., Sears, T.A., Yanez-Munoz, R.J. & McMahon, S.B. Cortical overexpression of neuronal calcium sensor-1 induces functional plasticity in spinal cord following unilateral pyramidal tract injury in rat. PLoS biology 8, e1000399 (2010).

41. Do, J.L., Bonni, A. & Tuszynski, M.H. SnoN facilitates axonal regeneration after spinal cord injury. PloS one 8, e71906 (2013).

42. Wong, L.F. et al. Retinoic acid receptor beta2 promotes functional regeneration of sensory axons in the spinal cord. Nature neuroscience 9, 243-250 (2006).

43. Wu, D. et al. Actions of neuropoietic cytokines and cyclic AMP in regenerative conditioning of rat primary sensory neurons. Experimental neurology 204, 66-76 (2007).
